# FRNA Bacteriophages as Viral Indicators of Faecal Contamination in Mexican Tropical Aquatic Systems

**DOI:** 10.1371/journal.pone.0170399

**Published:** 2017-01-23

**Authors:** Luis Jose Rene Arredondo-Hernandez, Carlos Diaz-Avalos, Yolanda Lopez-Vidal, Gonzalo Castillo-Rojas, Marisa Mazari-Hiriart

**Affiliations:** 1 Laboratorio Nacional de Ciencias de la Sostenibilidad, Instituto de Ecología, Universidad Nacional Autónoma de México, Mexico City, Mexico; 2 Departamento de Probabilidad y Estadística, Instituto de Investigaciones en Matemáticas Aplicadas y en Sistemas, Universidad Nacional Autónoma de México, Mexico City, Mexico; 3 Programa de Inmunología Molecular Microbiana, Departamento de Microbiología y Parasitología, Facultad de Medicina, Universidad Nacional Autónoma de México, Mexico City, Mexico; Natural Environment Research Council, UNITED KINGDOM

## Abstract

A particular challenge to water safety in populous intertropical regions is the lack of reliable faecal indicators to detect microbiological contamination of water, while the numerical relationships of specific viral indicators remain largely unexplored. The aim of this study was to investigate the numerical relationships of FRNA-bacteriophage genotypes, adenovirus 41, and human adenoviruses (HADV) in Mexican surface water systems to assess sewage contamination. We studied the presence of HADV, HADV41 and FRNA bacteriophage genotypes in water samples and quantified by qPCR and RT-qPCR. Virus and water quality indicator variances, as analyzed by principal component analysis and partial least squared regression, followed along the major percentiles of water faecal enterococci. FRNA bacteriophages adequately deciphered viral and point source water contamination. The strongest correlation for HADV was with FRNA bacteriophage type II, in water samples higher than the 50^th^ percentiles of faecal enterococci, thus indicating urban pollution. FRNA bacteriophage genotypes I and III virus indicator performances were assisted by their associations with electrical conductivity and faecal enterococci. In combination, our methods are useful for inferring water quality degradation caused by sewage contamination. The methods used have potential for determining source contamination in water and, specifically, the presence of enteric viruses where clean and contaminated water have mixed.

## Introduction

Waterborne enteric viruses inflict a heavy disease burden on developing countries. Enteric viruses negatively impact the quality of life for people and reduce their productivity and the number of days spent working. Enteric virus emissions to water bodies and the low infectious doses required represent a major obstacle to further mortality reductions in children younger than five years of age. The incidence of enteric viruses in children can be very high, as revealed by the presence of one viral agent in 43% of the children living in developing countries where most diarrhea attributable deaths occur, being rotavirus the most common pathogen in children, followed by norovirus in all countries [1]. Indeed, a relationship was found between the number of hepatitis cases and monsoons in India [2], thus highlighting the prominence of the waterborne route for enteric viruses. In addition, a relationship was found between the number of gastroenteritis outbreaks, and heavy rainfall and runoff events in the USA [3] and across the world [4]. Nevertheless, at least 50% of the gastroenteritis cases in the USA have an unknown causal agent [5].

It is widely accepted that inadequately treated wastewater and sewer discharges are the primary means by which enteric viruses gain entry to the environment via combined sewer overflows and/or cross connections [6]. It appears that urban conglomerates emit the highest number of viral particles of enteric virus to water bodies, and this finding is related to the size of a population, the number of people connected to the sewerage system, and the sewage treatment level [7]. Consequently, virus transmission through contact with contaminated water sources [8], such as effluent impacted recreational ponds or beaches [9,10], or via irrigation water carrying wastewater [11], is still a major water safety concern in many countries.

Much effort has been made to develop consistently good molecular indicators of bacteria or viruses to assist water quality assessment with improved sensitivity. However, such methods have not been systematically evaluated for use in tropical countries with medium incomes [6]. In such settings, evaluation of the sensitivity and accuracy of the various molecular indicators is helpful for determining water quality and source [12,13,14]. Successful day-to-day water quality monitoring relies on rapid molecular identification of waterborne pathogens and determination of their spatial-temporal distributions. Molecular quantification of viruses or bacteriophages is likely to be the only reliable method that is sufficiently fast to act as an early warning system to enable corrective action to be applied in a timely manner in regions with tropical environmental waters [15]. Therefore, we sought to assess the usefulness of multivariate principal component analysis (PCA) and partial least squared (PLS) regression as a descriptor of virological water quality and an indicator of contamination or service failure in Mexico, respectively. We employed PCA and PLS regression to explore the relationship between faecal enterococci and the presence of two types of commonly used molecular indicators, FRNA bacteriophages (genotypes I to III) and human adenoviruses (HADV), in four Mexican surface water systems. FRNA bacteriophage genotypes I to III have been consistently shown to be associated with sewage contamination of surface water [16,17,18]. Their presence in water is frequently correlated with the presence of enteric viruses in a number of water sources [19], while it is ever present in the water used for drinking water production [20].

Robust molecular methods for HADV (causative agents of respiratory infections and sporadic conjunctivitis [21]) and HADV41 (which causes up to 20% of the diarrhea cases in children under five years of age) detection/quantification have been widely applied and, in contrast to enterovirus qPCR, their number and concentration are statistically associated with effluent dominated waters [22]. In contrast, various water quality indicators have been analyzed according to the relative frequencies of faecal enterococci in a number of water matrices. The aim of this study was to investigate the numerical relationships of FRNA-bacteriophage genotypes, adenovirus 41, and human adenoviruses (HADV), in Mexican surface water systems to assess sewage contamination. All microorganisms referred in the manuscript are faecal indicators, while FRNA bacteriophages and adenovirus are also virus indicators as a cell host is needed for replication.

## Materials and Methods

### Experimental design

Multivariate analysis compared the following indicators: faecal enterococci, viruses, and the physicochemical variables controlling the structure of the data, according to the major faecal enterococci percentiles, defined as each 10 and/or 25 percentile break from maximum CFU/100 mL at Central Mexico sampling point. The strategy, allowed us to perform comparison regardless the water system origin of each sample (S1 Table). The physicochemical and bacteria-specific properties for FRNA bacteriophage genome groups I and III were assessed for 28 samples. In one third of the samples, FRNA bacteriophage group II, HADV, and the genome variants of HADV41, were also quantified by reverse transcription-polymerase chain reaction (RT-qPCR) and quantitative real-time polymerase chain reaction (qPCR) analyses.

### Water sources

Samples were collected from the following four Mexican surface water systems: the Magdalena River and the Cuitzmala River (natural systems), the Xochimilco altitude wetland (a semi-natural system mostly comprising a canal lentic system within Mexico City), and the Mezquital Valley (a man-made system). These four water systems, which represent the various types of water systems in Central Mexico and are located in the intertropical region of the country, have faecal contamination and conditions that facilitate the establishment of microbial niches. No specific permissions were required to enter these locations because they are natural bodies of water with open access. Field studies did not involve endangered or protected species The Magdalena River, which is a peri-urban system located in the area surrounding Mexico City, flows through a forested area and is used as a source for drinking water production. Upon entering the urban area, the river receives municipal wastewater discharges [23]. In contrast, the Cuitzmala River water system flows through a tropical area with a small population and is located on the coast in Jalisco State in Western Mexico [24]. The Xochimilco wetland is reminiscent of the Mexico basin lacustrine system [25]; it is a managed aquatic system recharged with treated wastewater and receives storm water during rainy season. In a connected distant area, agriculture is practiced in a region known as the Mezquital Valley (Hidalgo State, Mexico); here, a significant proportion of the municipal wastewater from Mexico City is discharged without treatment [26].

### Sample collection

According to the faecal enterococci colony counts determined previously, a total of either 1-L or 20-L water samples were collected in triplicate in autoclaved polypropylene bottles, and were then transported to the laboratory at 4°C for virus concentration (S2 Table). Next, the samples were processed following standard procedures within 6 h of collection as described by [27]. The 20-L water samples were concentrated by ultrafiltration with polycarbonate ultra-filters (Hemoflow F80A, Fresenius Medical Care, Waltham, MA, USA), using a perfusion rate of 1700 mL/min, as described by Hill et al. [28] while adenovirus detection required further concentration (10,000 X), using Amicon centrifugal units (30,000 MWCO; Merck Millipore, Billerica, MA, USA). Then, the samples were concentrated to a 200-mL final volume, and stored in 40-mL aliquots. Afterwards, RNA was extracted from the 100X concentrated sample to evaluate the FRNA bacteriophage genotypic groups present using RT-qPCR. Subsamples were frozen and stored at −70°C [29], after which the DNA was extracted.

### Water characterization

#### Physicochemical analyses

Physicochemical parameters such as dissolved oxygen, pH, electrical conductivity, and temperature, were determined for the four water systems, *in situ*. The following sensors were used: Rox optical dissolved oxygen (mg/L) sensor, pH 6561 sensor, temperature (°C), electrical conductivity (μS/cm), and total dissolved solids (mg/L) (YSI Inc., Yellow Spring, OH, USA). The sensors were calibrated according to the manufacturer’s instructions. The parameters were measured *in situ* using a YSI6600V2 water quality sonde (YSI Inc.).

#### Faecal *enterococcus* counts

Faecal enterococci counts were determined by the membrane filtration method 9230C, according to the 21^st^ edition of Standard Methods for the Examination of Water and Wastewater. Briefly: membrane filters (0.45 mm cellulose acetate, Millipore MF type HA) were placed on KF Streptococcus agar for streptococci/enterococci. Incubation was performed with a WTB Binder brand incubator at 35°C for 48 h according to APHA [27]. This is a quantitative method to determine the number of colony forming units (CFU) on a membrane, as described previously [27]. The bacterial counts were expressed as the base-10 logarithm of the number of CFU per 100 mL.

### Viral quantification

#### Extraction of viral DNA and RNA

Following the manufacturer’s instructions, viral nucleic acids were extracted from samples using QIAamp Viral RNA Mini Kit (Qiagen, Valencia, CA, USA) and QIAamp DNA Stool mini kit (Qiagen) for viral RNA and DNA, respectively. In addition to spin protocol in kit and the regular washing steps, glycogen co-precipitation was employed to remove any environmental inhibitors present in the samples while PCR inhibition was assessed by dilution series [29,30]. Finally all extracts were stored at −70°C until use.

#### Reverse transcription of FRNA bacteriophages

To obtain first strand in a two-step RT-qPCR, Superscript III first strand synthesis system was used according to manufacturer instructions, with minor modifications. After 5 min at 65°C and secondary structure elimination in a 5 μL solution containing 2μL RNA, and 1μL specific reverse oligonucleotides (2μM)—to match 3´end of specific FRNA genotype template sequence as described by Ogorzaly *et al*. (2006, 2007)—SuperScript III reverse transcriptase (Life technologies, Invitrogen, Carlsbad, CA, USA) was added, plus 1.5 μL MgCl_2_, (25mM), 1.5 μL DTT0.1M, and 1μL10x RT-buffer [31,32]. Next, cDNA-reverse transcriptase product was synthesized at 50°C for 1 h in a thermocycler (GeneAmp PCR system 9700, Life technologies, Applied Biosystems, Foster City, CA, USA). Finally, the enzyme was heat inactivated at 95°C for 5 min.

### Quantification of HADV, HADV41 and FRNA bacteriophage genotypes I to III by qPCR and RT-qPCR

#### Standards, RT-qPCR and qPCR curves for the viral indicators

Bacteriophage MS2 (ATCC 15597-B1), FRNA genotype I (GI), and Qβ (ATCC 23631-B1) FRNA genotype III (GIII), were cultured from stock vials purchased from American Type Culture Collection (ATCC, http://www.lgcstandards-atcc.org/). Propagation took place using *Escherichia coli* host (ATCC 23631) culture during log phase, as in ISO method 10705–1. FRNA genotypes GI, GII, and GIII RNA fragments, were RT-PCR amplified, sequenced, and subjected to GenBank database Basic Local Alignment Search Tool analysis after plasmid construction to ensure high sequence identity homology to FRNA bacteriophage and enteric virus specific sequence [33]. RNA, extracted and converted into cDNA was cloned into a pCR2.1 TOPO vector (TOPO TA Cloning Kit, Invitrogen Life Science Technologies), following manufacturer instructions. HADV and HADV41 DNAs were purchased from ATCC VR-930D. Specific endpoint PCR DNA amplicons were subsequently cloned into a plasmid vector (pCR2.1-TOPO) using the one-shot chemical transformation method described by the manufacturer (TOPO TA Cloning Kit for Sequencing). The plasmid construct was column-purified and adjusted to 2×10^8^ copies per μL for use as a standard stock solution for qPCRs. Standards were diluted to 10^1^–10^5^ copies per qPCR reaction of standard curve to calibrate the concentrations of the target gene detected in the FRNA genotype. The primers and minor groove binding probes originally designed by Ogorzaly [31,32] were synthesized by Applied Biosystems; 500 nmol of each primer and 250 nmol of each hydrolysis probe were used for the monoplex assays. Amplification curves for HADV and HADV41 were constructed for genome quantification as previously described [34,35,36] (Table 1).

**Table 1:** Primers and probes used for RT-qPCR and qPCR genome quantification of the virus indicators used in this study.

Viral indicators	Primers and probes	Sequence	Author	Reference
FRNA bacteriophage genotype				
Group I	GI forward	5´-TCGATGGTCCATACCTTAGATGC-3´	Ogorzaly *et al*.	[31, 32]
	GI reverse	5´-ACCCCGTTAGCGAAGTTGCT-3´		
	Probe for GI	FAM-CTCGTCGACAATGG-MGBNFQ		
Group II	GII forward	5´-TGCAAACCTAACTCGGAATGG-3´		
	GII reverse	5´-AGGAGAGAACGCAGGCCTCTA-3´		
	Probe for GII	FAM-TCCCTCTATTTCCTC-MGBNFQ		
Group III	GIII forward	5´-CCGCGTGGGGTAAATCC-3´		
	GIII reverse	5´-TTCTTACGATTGCGAGAAGGCT-3´		
	Probe for GIII	FAM-AAGCGGGTGCAGTT-MGBNFQ		
Human Adenovirus				
HADV	JTVXF	5´-GGACGCCTCGGAGTACCTGAG-3´	Jothikumar *et al*.	[34]
	JTVXR	5´-ACIGTGGGGTTTCTGAACTTGTT-3´		
	JTVXP probe	FAM-CTGGTGCAGTTCGCCCGTGCCA-BHQ		
Genotype 41	HAdV-F4041-hex157f	5´-ACCCACGATGTAACCACAGAC-3´ CACTTTGTAAGAATAAGCGGTGTC	Jiang *et al*., modified by Xagoraraki *et al*.	[35] [36]
	HAdV-F41-hex246r	5´-CACTTTGTAAGAATAAGCGGTGTC-3´		
	probe HAdV-F4041-hex214rprobe	FAM-CGACKGGCACGAAKCGCAGCGT-BHQ-1		

FAM = 6-Carboxyfluirescein. MGB-NFQ = Minor Groove Binder moiety-Nonfluorescent quencher. BHQ = Black Hole Quencher. I = Inosine. K = G + T.

For each HADV, a qPCR was performed in a 20-μL volume with 10 μL TaqMan Master Mix, 1 μL each of 10 μM forward and reverse primers, 0.6 μL 10 μM hydrolysis probe, and 5 μL of a DNA sample or standard. The thermal profile consisted of one cycle at 95°C for 15 min, followed by 45 cycles at 95°C for 15 s, 55°C for 33 s, and 72°C for 33 s. The qPCR program for HADV41 consisted of 15 min at 95°C, followed by 45 cycles at 95°C for 15 s, 60°C for 33 s, and 72°C for 33 s. The FRNA genotypes were amplified using the following program: 15 min at 95°C, followed by 45 cycles at 95°C for 15 s and 60°C for 60 s. All the samples were amplified using a 7500 Real-Time PCR System (Life technologies Applied Biosystems). Each sample (i.e., viral DNA extracts or cDNA reverse transcripts) and standard controls were run in triplicate. All bacteriophage and virus quantitation by qPCR included a negative control reaction mixture (PCR-grade H_2_O without template).

### Statistical analysis

The concentrations of the microorganisms were log10-transformed and linearity was assessed using Excel 2010 software (Microsoft Corporation, Redmond, WA, USA). Multivariate statistical analyses were performed using XLSTAT software version 13.4.03 (Addinsoft, France). Sample data were ordered into major percentiles according to the faecal enterococci water quality logarithm. Parallels in the enteric virus indicators along the faecal enterococci percentiles were examined by PCA and factor analysis to explore the variable physicochemical relationships between the pollution hypothesis and molecular indexes (FRNA groups I, II, and III, HADV and HADV41) for the sampling points. Finally, the most important variables identified by PLS regression—recommended regression method when collinearity is expected—included samples around each faecal enterococci major percentile and uphold PCA when low sample number [37,38].

## Results

### Viral index: FRNA bacteriophages, HADV and genotype 41

**Fig 1:**
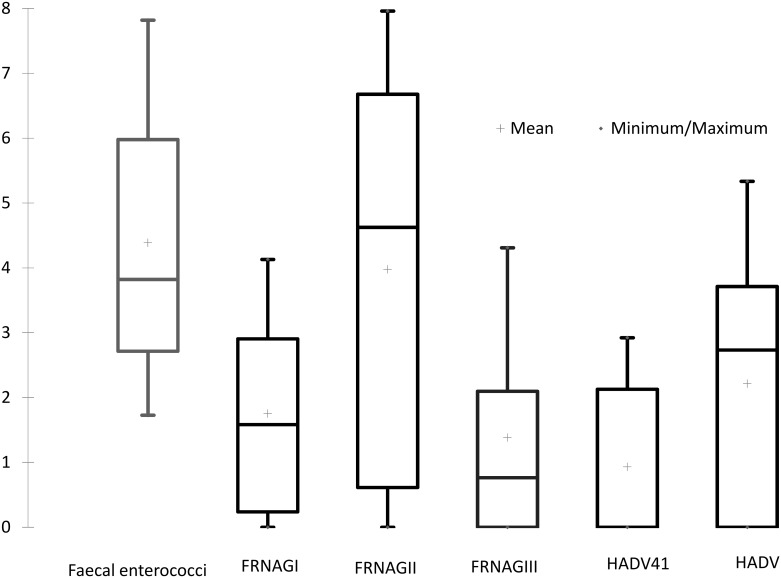
Log number of the distribution of faecal indicators from tropical water samples.

FRNA genotype I, II, and III bacteriophages were detected and quantified by RT- qPCR (Fig 1). FRNA genotypes I and III had similar values, unlike FRNA genotype III (FRNAGIII), which was predominant over FRNA genotype I in most of the water quality faecal enterococci log percentile groups, especially those higher than the 50^th^ percentile. The proportions were not constant in all of the water samples. FRNAGIII was not detected in any samples from the Cuitzmala River; however, there were fewer bacteriophages present in the sampling points from this river, compared with samples from the same faecal enterococci percentile. The genome numbers were higher for FRNA bacteriophages within the 10^th^ faecal enterococci percentile samples because of the sample origin: wastewater filtered through soil that emerged at the Cerro Colorado Spring, in the Mezquital Valley.

FRNA bacteriophage genotype II genome counts consistently outnumbered those of FRNA genotypes I and III and were nearly twice the FRNA genotype I log genome count. The minimum number of positive enterococci samples for HADV (HADV = 1.82 log genomes/100 mL) and HADV41 (ADV41 = 2.73 log genomes/100 mL) was 3.76 logFE. The ADV41/HADV ratio ranged from 0.76 in the semi-urban agricultural area to 0.66 near the treated wastewater point source in the Xochimilco tropical high-altitude wetland. The ratio of 0.54 in the Magdalena River urban area indicates that ADV41 represented at least half of the total number of HADVs. No samples showed PCR amplification inhibition.

**Fig 2:**
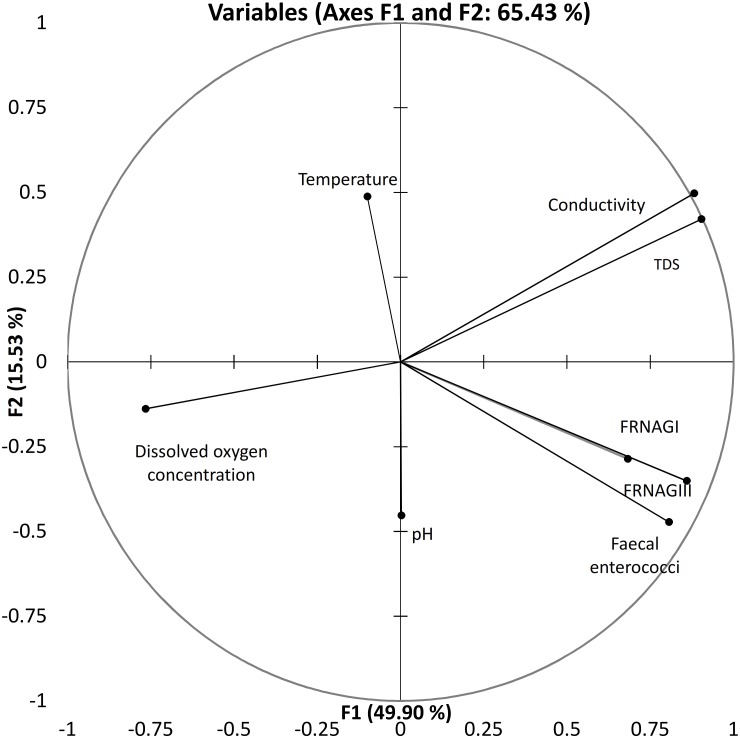
Principal component analysis bi-plot showing dispersion of vector physicochemical parameters, faecal enterococci, and FRNA bacteriophages I and III among tropical water samples.

PCA of 28 samples covering the whole spectra of water quality that we assessed resulted in 65% of the variance explication by the first two components. They showed major loadings of conductivity and dissolved oxygen variables, followed by FRNA genotype I and III loadings to PCA. Faecal enterococci constituted the less explanatory variable. PCA arranged the eight variables studied herein into two clusters (Fig 2). FRNA bacteriophage genotype I, genotype III, and faecal enterococci variables formed a cluster while conductivity and total dissolved solids separated from the rest of the variables. All the variables seemed to be anti-correlated to the dissolved oxygen variable coordinate loadings. The temperature variable vector on the coordinate plane was perpendicular to that of dissolved oxygen.

**Fig 3:**
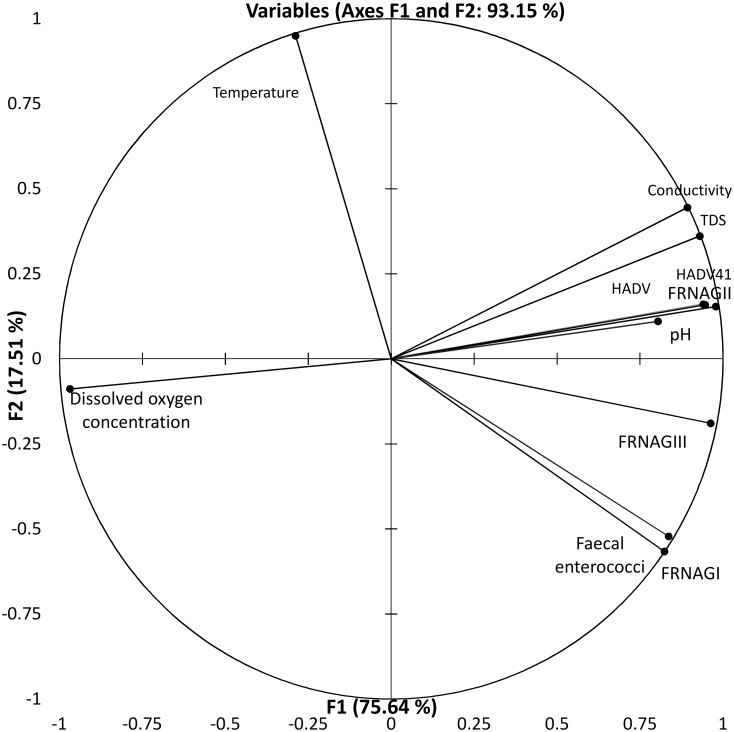
Principal component analysis bi-plot showing the vector proximity for the physicochemical, faecal enterococci, FRNA bacteriophages genotype II, human adenovirus, and adenovirus 41 among selected tropical water samples.

**Fig 4:**
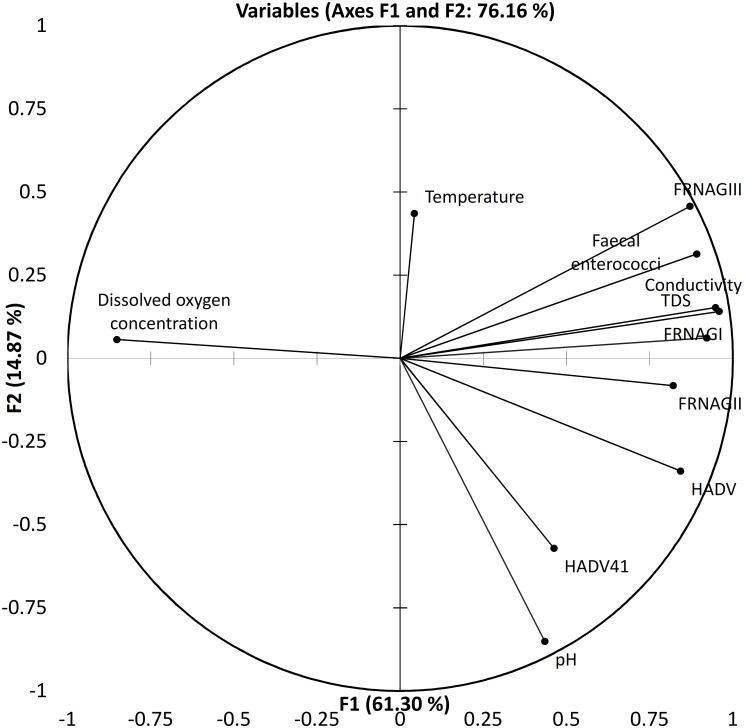
Principal component analysis bi-plot showing the vector proximity for the FRNA bacteriophages genotypes, along with the reduced variance for human adenovirus and human adenovirus 41 in higher than 50^th^ percentile faecal enterococci percentiles in the tropical water samples.

PLS regression models for FRNA bacteriophage genotype I and III genome counts showed that dissolved oxygen and temperature are the most important variables explaining genotype I. However, dissolved oxygen concentration is also a significant variable for the FRNA genotype III genome count in PLS regression, conductivity, and faecal enterococci variables. It also adjusts the model of complete composite of samples (S1 and S2 Figs). Factor analysis and PLS regression for each of the major faecal enterococci deciles across the different sample origins showed that at least 76% of the variance was explainable and the percentage was higher if the samples belonged to the same aquatic system. The first decile, the most oxygenated one, showed FRNA bacteriophage genotype I and conductivity variable vectors parallel to each other. It also showed that GIII was the closest to faecal *enterococcus*. However, the proximity of FRNA bacteriophage genotype I and/or III to faecal enterococci varied along the major percentile sample clusters included in this analysis. The PLS regression showed that the FRNA genotype III was mostly associated with those surface water samples taken downstream of a waste water treatment plant or sewerage input; however, the segregation of the data was not absolute. Therefore, a subset composed of a third of the samples representing the water quality spectra of the whole set was assessed to include HADV, HADV41, and FRNA genotype II viral indicators. We repeated the multivariate analysis for the complete subset (Fig 3) and for the faecal enterococci cluster samples at the 50^th^ percentile (Fig 4). Either 76% or 93% of the variance was explained by the first two factors of the analysis, from which FRNAGI, conductivity, and total dissolved solids had the highest nominal contributions followed by FRNAGII and FRNAGIII. Regardless of the number of samples included, dissolved oxygen was anti-correlated to the rest of the variables. FRNA bacteriophage genotype II seems to best characterize decay in the dissolved oxygen concentration variable, because both follow almost the same continuous line in the plot (Fig 3). Variable vectors describe a fan shape through the second and the third Cartesian plane quadrants, giving a mirrored image with pH, virus indicators and FRNA bacteriophage genotype II in the (positive, negative) quadrant. The temperature variable vector ran parallel to the Y axis (F1), while the counts for the FRNA bacteriophage genotype III genome variable and the faecal enterococci variable had the highest values on both axes and were located next to each other on the plane. FRNA bacteriophage genotype I was more closely associated with conductivity as determined from the proximity of these items on the plot, and in the representative subset it was only associated with faecal enterococci in the higher than 50^th^ percentile area of the plot. Multivariate analysis of the higher than 50^th^ percentile samples rendered similar results. However, the anti-correlation for the dissolved oxygen variable to FRNA bacteriophage genotype II and the correlation between HADV, HADV41 and FRNA bacteriophage genotype II genome number variables were strengthened. Respective loadings of FRNA bacteriophage genotypes I and III graphically locate the genotype I vector besides the faecal enterococci vector, while genotype III is located halfway between faecal enterococci and the human-specific HADV and HADV41 viral indicators.

## Discussion

Molecular tools for accurate quantification of the biological indicators of contamination with waterborne enteric viruses are urgently needed for timely alerts of water safety breaches in populated areas of tropical countries [37]. Incidental transmission of enteric viruses via recreational bathing water and contaminated drinking water poses a real threat to public health because of the large number of enteric viruses emitted, wastewater usage in agriculture, inadequate wastewater treatment, infrastructure maintenance, and operational deficiencies [38,39,40]. Consistently, urban rivers are receiving higher loads of faecal contamination [41,42], becoming part of sewer system. In wastewater, a highly variable amount of bacterial indicators has been reported [43]; as for suspended particle size, concentrations reaching as high as 10^8^/100 mL have been reported at Mezquital Valley [44]. Differentiating non-point contamination to which enteric viruses don’t correlate at all has been a major challenge [45,46]. Incoming from animal sources, and indicator habituation to water environment may exert a different risk than CSO effluent discharge. Similarly, unpredictable heavy rainfall can also increase virus emissions into water sources, but only up to 20% of bacterial indicators are from wastewater [47]. Therefore, where practicable, corrective measures should be employed to avoid the unintentional mixing of recreational water and source water used for potable water supply with virus-contaminated sewer overflows, sewage, or effluent from wastewater treatment plants. However, ensuring that these waters remain separate requires indicator monitoring systems that overcome the specific limitations of monitoring bacteria, particularly in tropical middle-income and developing countries. Accurate and reliable identification of point and diffuse contamination in a water system requires an indicator that can provide information on the contamination point in time and space, be sensitive enough to identify trace amounts of wastewater, and be fast enough to quantify the bacterial indicator to avoid a service safety failure, thereby limiting the risk of enteric virus spread to the population to an acceptable threshold.

In the present study, FRNA bacteriophages absolute genomes were detected by RT- qPCR amplification of conserved sequences using primer sets previously probed in water environment assessments. Quantifiable amounts of specific product were detected in samples from the four Mexican water systems (the Magdalena and Cuitzmala natural river systems, the semi-natural Xochimilco altitude wetland system, and the man-made Mezquital Valley system) that were examined (S5–S12 Figs). Multivariate analysis was used to compare the data obtained for the faecal enterococci and indicator viruses and the physicochemical variables controlling the structure of the data. Follow-up of the molecular indicator variance through the gradient on the graph showed a wide area where the viral indicators were concentrated (Figs 3 and 4), which narrowed as the faecal percentile increased. Above the 50^th^ percentile mark, mainly including the sewerage input sampling points, we found that HADV, gastroenteritis-related genotype 41, and FRNA genotype II, behaved in a similar fashion. However, as with other studies that have attempted to find a single indicator that can precisely explain the presence of enteric virus, it is clear that as the system becomes more complicated and contains a lower than 50^th^ percentile value, pollution hypothesis should be interpreted cautiously in a site by site analyses. Although, the vector representing FRNA bacteriophage genotype II is closer to HADV vector than any other, the long distance between them reflects the fact that detection in the same samples is not as common, neither is it for ADV41. We conclude that detection of complete set of molecular indicators could be used as a precautionary measure for accurate discrimination between wastewater and wastewater mixtures [33,48].

Previous studies [12,18] measuring the absolute number of genomes for various molecular indicators have reported similar results to our own (S3 and S4 Figs). However, the diversity of water origin within a wide water quality spectrum allows a number of conclusions to be drawn from the relationships of the water quality variables and the molecular virus indicators.

As well as quantifying the levels of various viral indicators, we also conducted *in situ* measurements of various physicochemical parameters such as dissolved oxygen, pH, electrical conductivity, and temperature, in the four water systems. Dissolved oxygen was unique in showing an inverse correlation with the other parameters we measured, particularly the molecular indicators of viruses. This behavior might be partly explained by the role of oxygen in virus inactivation [41,49]; otherwise it just shows the inverse relation between oxygen concentration and consuming organic matter, which is mostly in particulate form and aggregate virus particles [50]. Nevertheless, the FRNA bacteriophage genotype II genome number increment clearly showed water quality degradation within the 50^th^ percentile for faecal enterococci and subsequent samples of examined set, and was also associated closely with HADV and HADV41 genome counts. Such a relationship between viral indicators has been reported previously [12] for river water that receives wastewater. However, the extent to which the relationship is maintained has now been determined. When the water samples under the 50^th^ percentile were included in the PCA analysis, the samples containing HADV were potentially identified by a combination of pH, conductivity, and FRNA genotype II interval values, all of them known indicators of wastewater abrupt water quality change.

In our analyses, the position of the FRNA bacteriophage genotype I values over the 50^th^ percentile is nearest to the faecal enterococci genome number; hence, there is no advantage in using this genotype to indicate the presence of virus in comparison with genotypes II and III. In the lower percentiles, FRNA bacteriophage genotype I and conductivity coordinates approached each other, probably reflecting a slow sustained increment of ions caused by weathering and non-point sources [42]. In consequence, there was a conductivity increase, concurrent with the extended persistence of FRNA bacteriophages genotype I in contact with their host in old [43] rather than fresh wastewater input. Even so, it seems that with the FRNA bacteriophage genotype III, which is less resistant than FRNA genotypes I and II, there is a loss of ability to indicate the presence of HADV, as it is probably removed from the highest percentiles being the most distant in the lowest percentiles. Nonetheless, this genotype is associated with faecal enterococci in mixed non-point sources. Like in Vergara, et al., (2015), the media of FRNA GIII number of genomes was quite below that of genotypes I and II [18]; however, media of genotype II outnumbered that of genotype I, probably due to urban wastewater predominance at most of the sampling points. Detailed analysis of FRNA bacteriophage genotype III genomic variation has shown its specific association with wastewater or animal sources [44]; however, this distinction was beyond the scope of this study.

A limitation of this study was the small number of water samples in the first deciles that capture the transitory nature of the emissions [45]; this resulted in an incomplete description of the virus distribution. Secondly, since samples were obtained from various aquatic systems, the results should not be extrapolated a priory, but advantages of each molecular indicator, should be addressed locally to suit specified water quality use. However, use of biological indicators as proxy measures for the presence of enteric viruses and water quality breaches in Central Mexico is supported by FRNA bacteriophage genomes being detected in spring water used as a source for the production of potable water in the Mezquital Valley area. In conclusion, Molecular indicator quantitation combined with multivariate statistical analyses showed functionality at inferring water quality degradation caused by sewage contamination. FRNA bacteriophage genotypes II and III, as well as HADV, can be used as indicators as long as the faecal enterococci numbers are above the 50^th^ percentile in mixed waters contaminated with sewage. These indicators may provide a useful early warning system for the contamination arising from sewage sources in water systems, or for recent contamination via virus emission, particularly in tropical water environments that are affected by enteric pathogens. Looking ahead, implementation of molecular indicators for proxy measurement of virus pollution in fresh wastewater to bring about reductions in the transmission of waterborne enteric bacteria and viruses in middle-income countries is technically feasible.

## Supporting Information

S1 Table Sampling points geographic coordinates. (DOCX)

S2 Table Amplification profile of qPCR of FRNA bacteriophages and adenovirus from environment water bodies. (DOCX)

S1 Fig Dendrogram showing similarities in the higher than 50^th^ percentile values for the faecal enterococci percentiles in tropical water samples. (TIF)

S2 Fig Cumulative distribution of faecal enterococci among representative samples. (TIF)

S3 Fig Fecal indicator concentrations at sampling points in Central Mexico. (PNG)

S4 Fig Fecal indicator concentrations at selected sampling points in Central Mexico. (PNG)

S5 Fig Amplification profile of RT-qPCR for FRNA bacteriophages GI from RNA extracted from waste water. Amplification plot.
(TIF)

S6 Fig Amplification profile of RT-qPCR for FRNA bacteriophages GI from RNA extracted from waste water. Standard curve.
(TIF)

S7 Fig Amplification profile of RT-qPCR for FRNA bacteriophages GII from RNA extracted from waste water. Amplification plot.
(TIF)

S8 Fig Amplification profile of RT-qPCR for FRNA bacteriophages GII from RNA extracted from waste water. Standard curve.
(TIF)

S9 Fig Amplification profile of RT-qPCR for FRNA bacteriophages GIII from RNA extracted from waste water. Amplification plot.
(TIF)

S10 Fig Amplification profile of RT-qPCR for FRNA bacteriophages GIII from RNA extracted from waste water. Standard curve.
(TIF)

S11 Fig Amplification profile of qPCR for human adenovirus DNA extracted from waste water. Amplification plot.
(TIF)

S12 Fig Amplification profile of qPCR for adenovirus genotype 41 DNA extracted from waste water. Amplification plot.
(TIF)
