# Supplementary material for: FRNA Bacteriophages as Viral Indicators of Faecal Contamination in Mexican Tropical Aquatic Systems
Source: PLoS One. 2017 Jan 23;12(1):e0170399. doi: 10.1371/journal.pone.0170399 (PMC5256921; doi:10.1371/journal.pone.0170399)
Supplement: S1 Table — (DOCX) [file pone.0170399.s001.docx]

Table S1. Geographic positions of the sampling points by aquatic system and log faecal enterococci percentiles.

| Sampling point | Latitude | Longitude | Water system | log10 faecal enterococci | Hierarchic ascendant classification | Percentile group |
| --- | --- | --- | --- | --- | --- | --- |
| an4 | 19.31974 | −99.22005 | Magdalena River | 2.45 | 1 | 10 |
| an5 | 19.29971 | −99.2515 | Magdalena River | 2.57 | 1 | 25 |
| an6 | 19.26548 | −99.29395 | Magdalena River | 2.96 | 1 | 25 |
| an7 | 19.28983 | −99.26456 | Magdalena River | 3.26 | 1 | 25 |
| zu3 |  |  | Magdalena River | 3.40 | 1 | 25 |
| zu5 | 19.30337 | −99.23725 | Magdalena River | 4.13 | 2 | 50 |
| zu9 | 19.31448 | −99.2214 | Magdalena River | 3.07 | 2 | 25 |
| zu10 | 19.31967 | −99.21995 | Magdalena River | 4.61 | 2 | 50 |
| zu11 | 19.29966 | −99.25152 | Magdalena River | 6.59 | 3 | 50 |
| Cuemanco | 19.2831678 | −99.1024454 | Xochimilco wetland | 3.77 | 2 | 50 |
| Santisima | 19.2617905 | −99.0926971 | Xochimilco wetland | 4.30 | 3 | 50 |
| chinampa experimental | 19.2745763 | −99.0967394 | Xochimilco wetland | 3.87 | 3 | 50 |
| Tezhuiloc | 19.2741472 | −99.0874697 | Xochimilco wetland | 3.93 | 2 | 50 |
| San Gregorio | 19.2621602 | −99.0476666 | Xochimilco wetland | 4.07 | 2 | 50 |
| eca | 19.8032146 | −104.71741 | Cuitzmala River | 2.53 | 3 | 25 |
| villa | 19.3842504 | −104.973982 | Cuitzmala River | 1.73 | 3 | 10 |
| San miguel | 19.6833627 | −104.737924 | Cuitzmala River | 2.70 | 3 | 25 |
| Jirosto | 19.7555363 | −104.754138 | Cuitzmala River | 2.52 | 3 | 25 |
| vena 2 | 19.3768324 | −105.007761 | Cuitzmala River | 1.86 | 2 | 25 |
| Tempisque | 19.5751823 | −104.802952 | Cuitzmala River | 2.01 | 2 | 10 |
| rio 2 | 19.3761183 | −104.989354 | Mezquital Valley | 1.95 | 3 | 10 |
| pbchl | 20.19221 | −98.54324 | Mezquital Valley | 7.62 | 4 | 75 |
| licu | 19.44162 | −99.04739 | Mezquital Valley | 7.82 | 4 | 75 |
| salto | 20.06308 | −99.24684 | Mezquital Valley | 7.70 | 4 | 75 |
| end ent | 20.05599 | −99.34003 | Mezquital Valley | 8.09 | 5 | 95 |
| endsal | 20.06307 | −99.24679 | Mezquital Valley | 8.07 | 5 | 95 |
| ccol | 20.056001 | −99.340001 | Mezquital Valley | 0.40 | 2 | 10 |
| piez1 | 20.12854 | −99.211001 | Mezquital Valley | 7.28 | 5 | 75 |
