# Supplementary material for: FRNA Bacteriophages as Viral Indicators of Faecal Contamination in Mexican Tropical Aquatic Systems
Source: PLoS One. 2017 Jan 23;12(1):e0170399. doi: 10.1371/journal.pone.0170399 (PMC5256921; doi:10.1371/journal.pone.0170399)
Supplement: S2 Table — (DOCX) [file pone.0170399.s002.docx]

| **Detector** | **Slope** | **Intercept** | **R2** | **Limit of**  **detection (fg)** | **Dynamic range** |
| --- | --- | --- | --- | --- | --- |
| **ADVH** | **-3.758874** | **46.970768** | **0.98634** | **2fg** | **20fg-200pg** |
| **adv41** | **-3.179571** | **34.551502** | **0.992983** | **2fg** | **20fg-200pg** |
| **FRNAGI** | **-3.469521** | **39.882114** | **0.994887** | **2fg** | **20fg-200pg** |
| **FRNAGII** | **-3.557347** | **47.73193** | **0.995316** | **2fg** | **20fg-200pg** |
| **FRNAGIII** | **-3.517509** | **42.449028** | **0.982401** | **2fg** | **20fg-200pg** |
