# Supplementary figures and images for: FRNA Bacteriophages as Viral Indicators of Faecal Contamination in Mexican Tropical Aquatic Systems
Source: PLoS One. 2017 Jan 23;12(1):e0170399. doi: 10.1371/journal.pone.0170399 (PMC5256921; doi:10.1371/journal.pone.0170399)

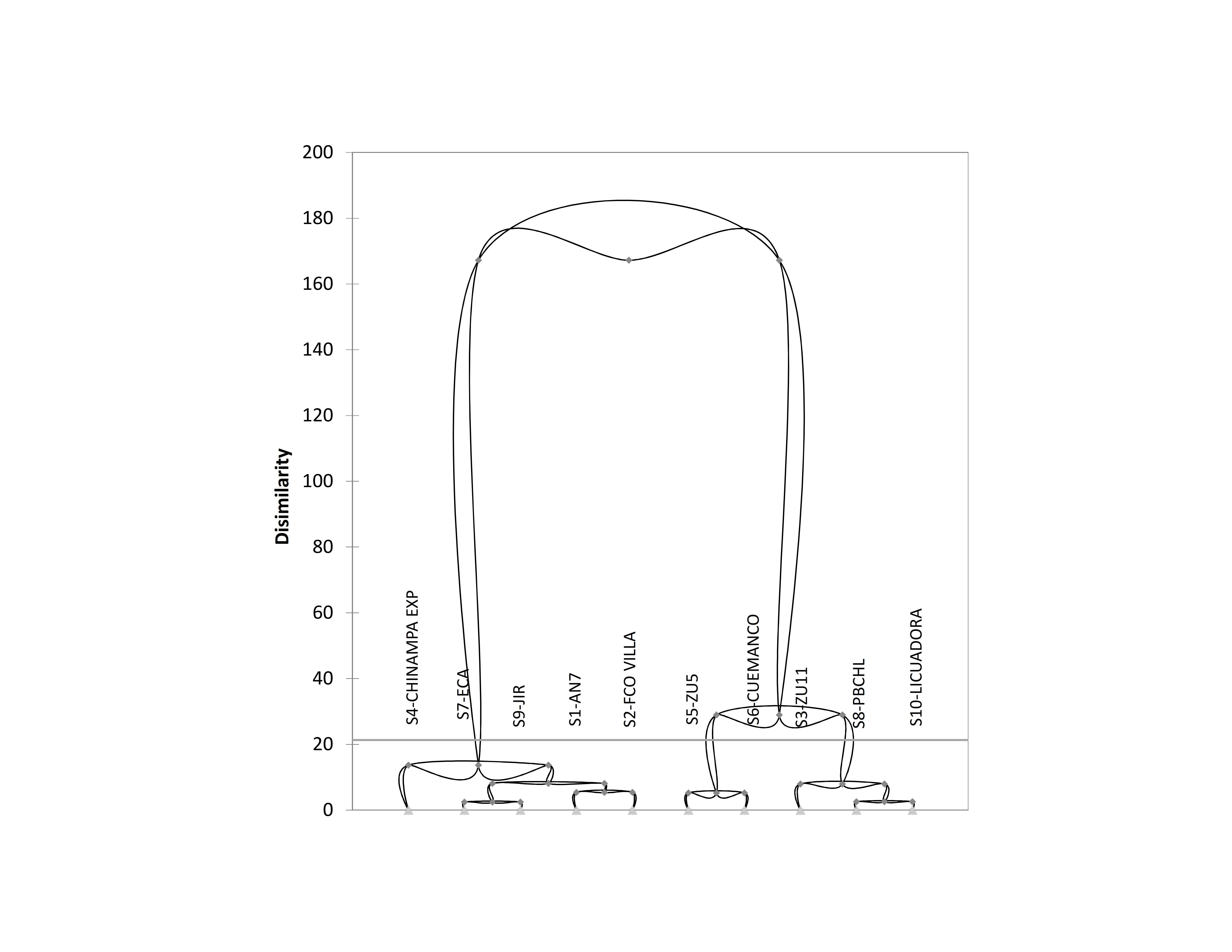

Supplement: S1 Fig — (TIF) [file pone.0170399.s003.tif]

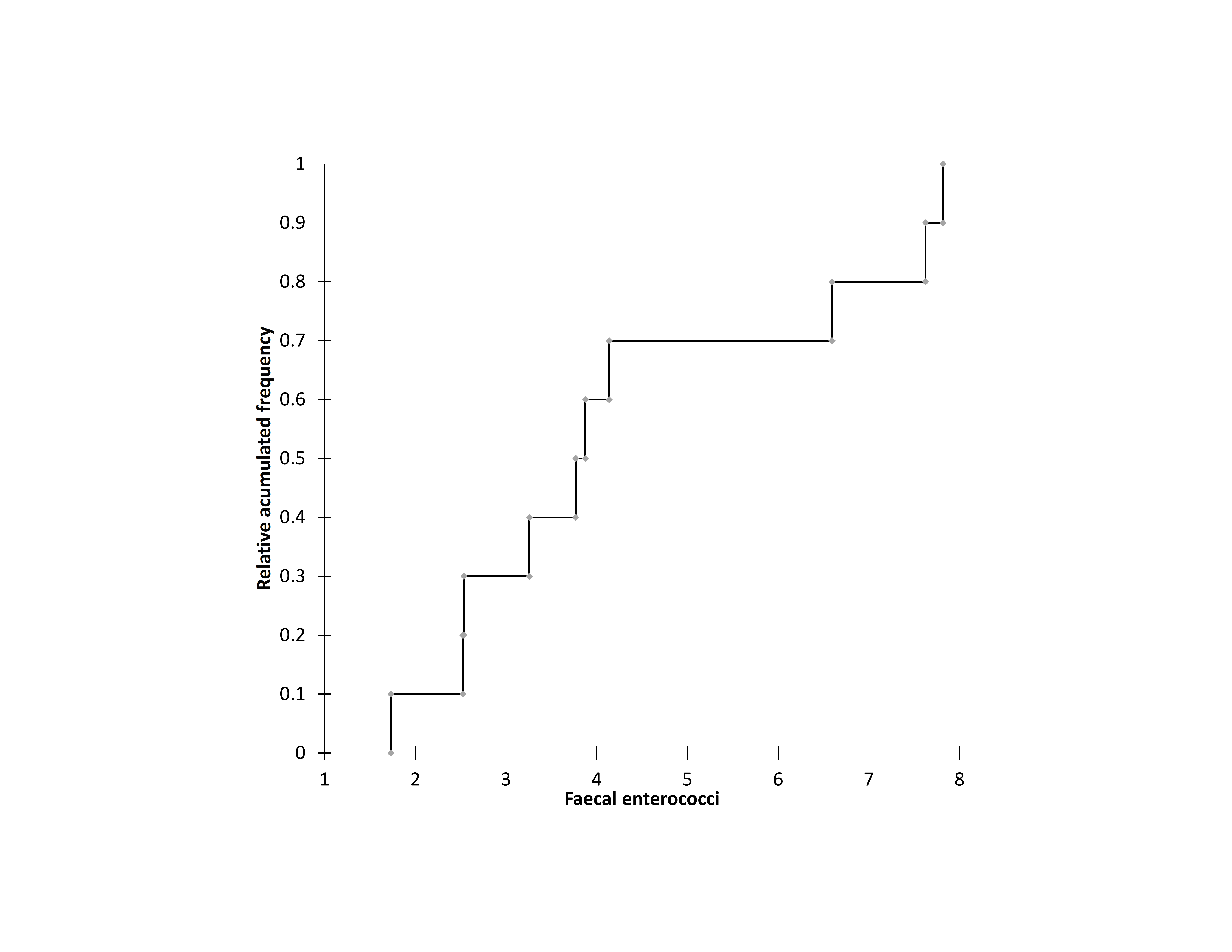

Supplement: S2 Fig — (TIF) [file pone.0170399.s004.tif]

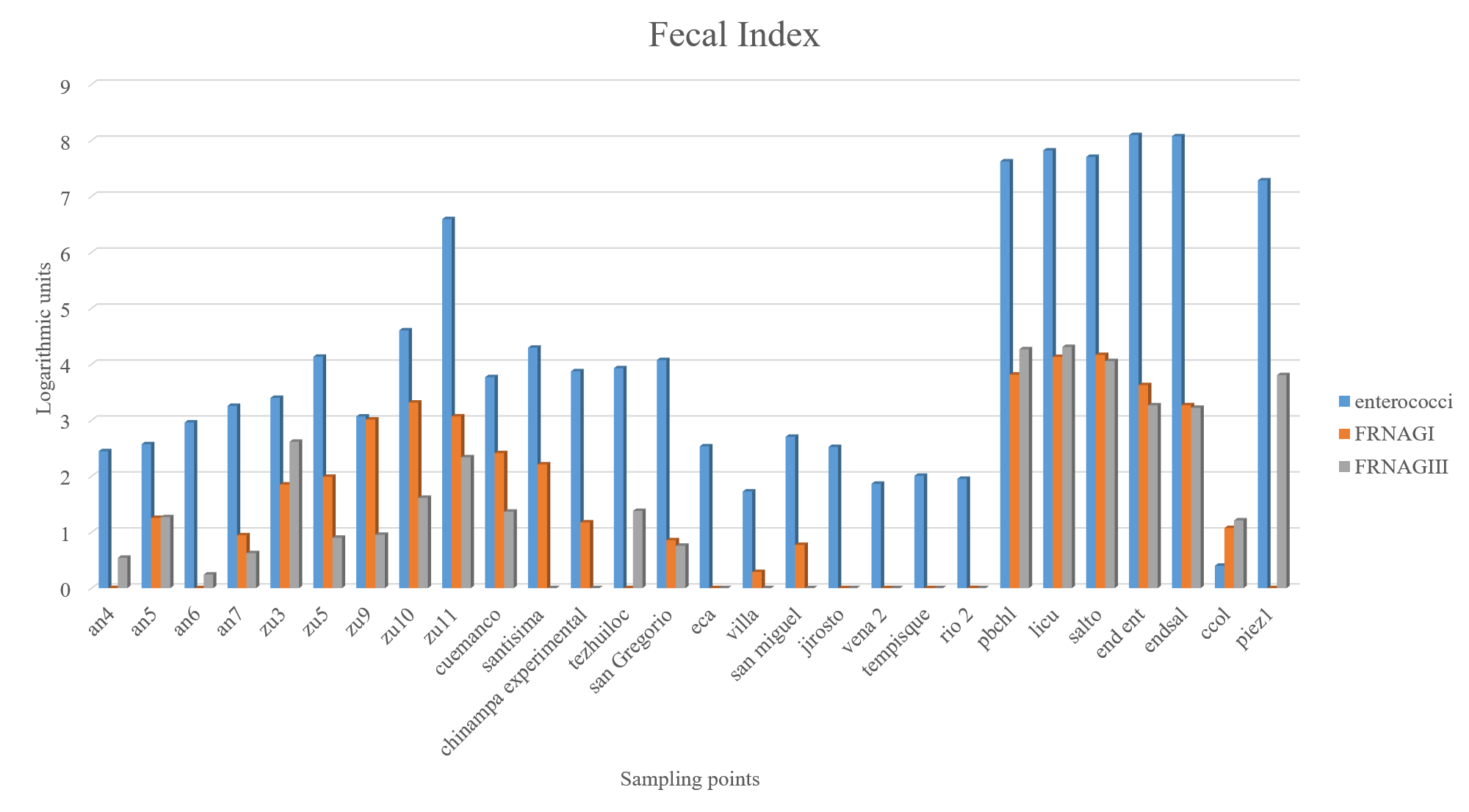

Supplement: S3 Fig — (PNG) [file pone.0170399.s005.png]

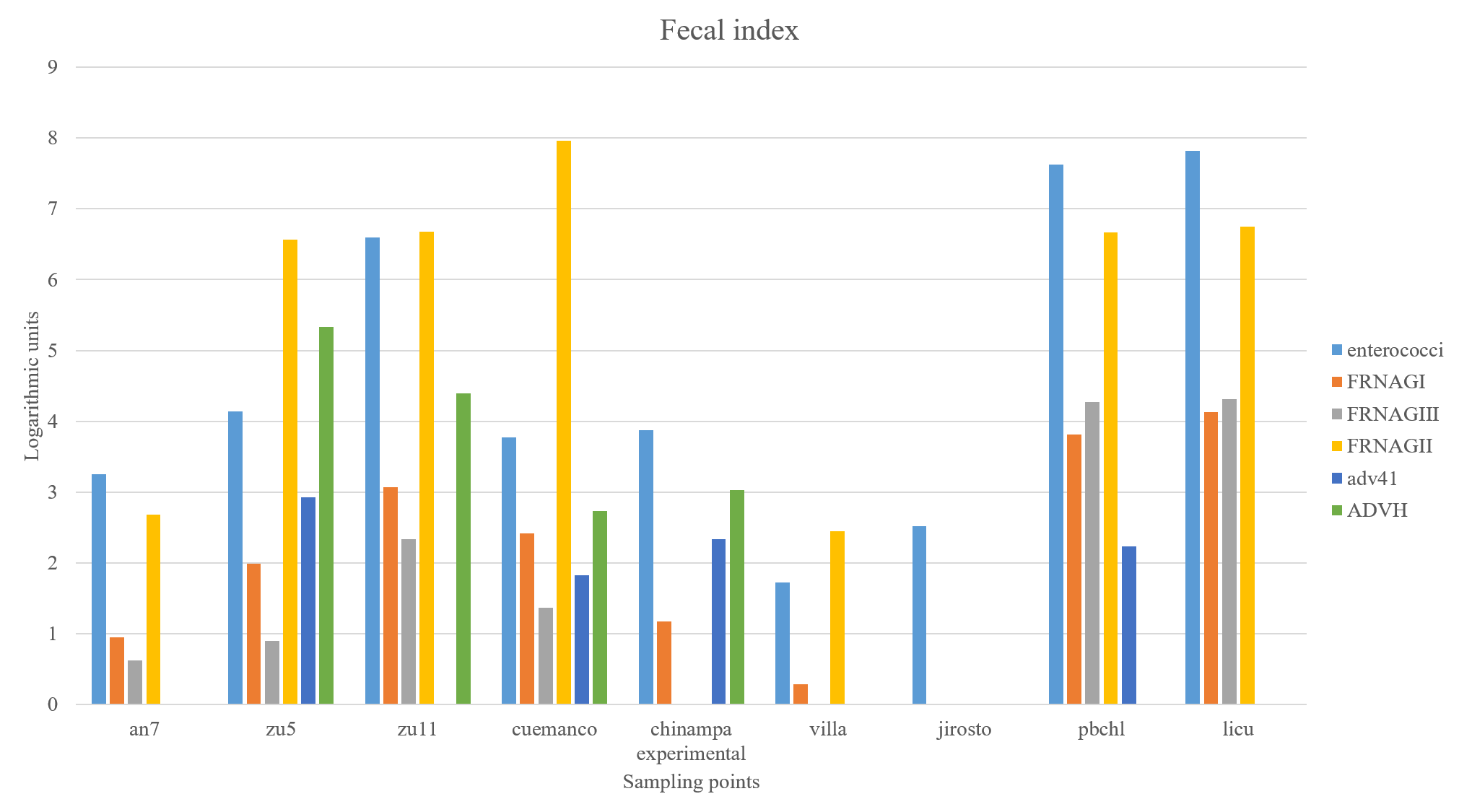

Supplement: S4 Fig — (PNG) [file pone.0170399.s006.png]

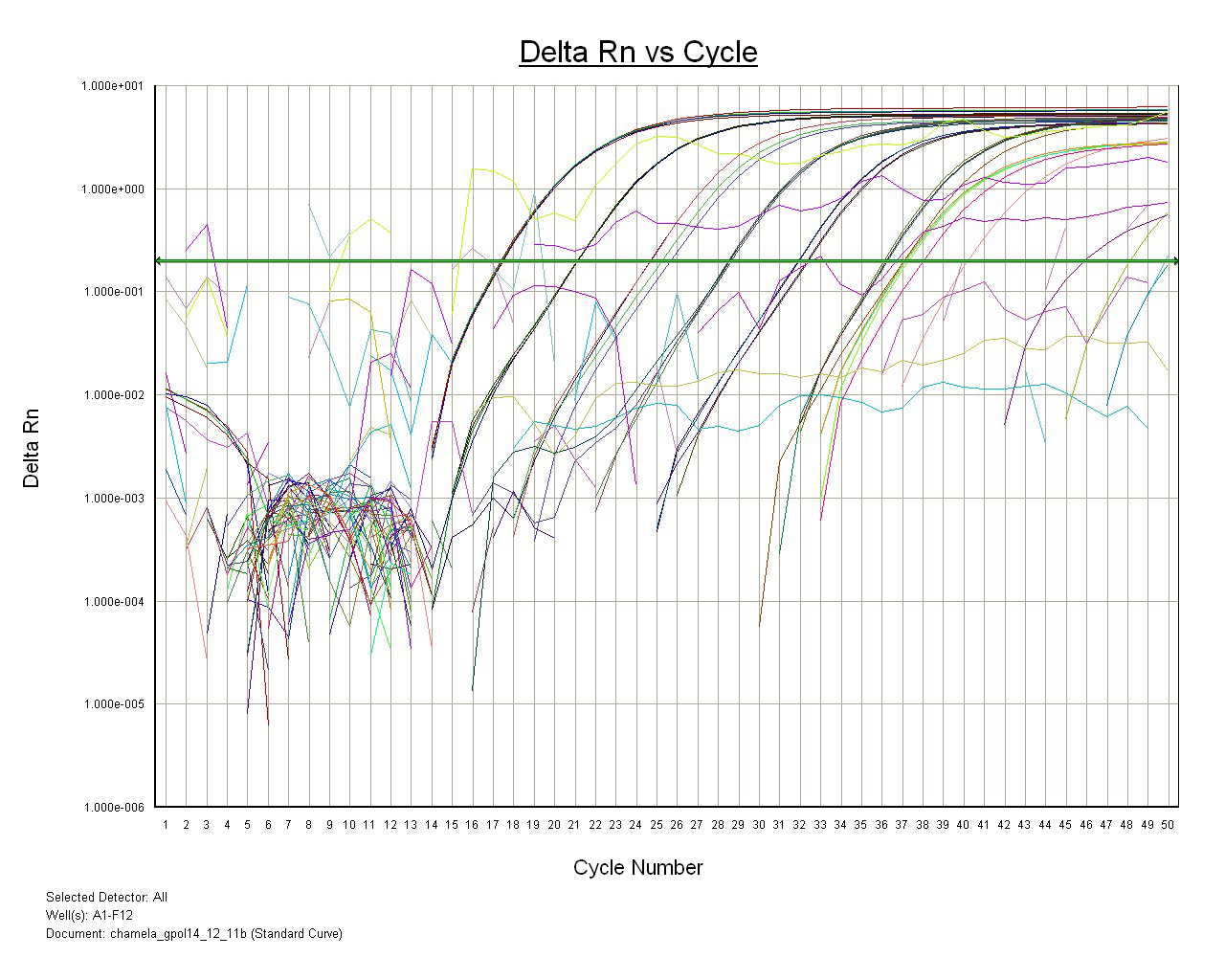

Supplement: S5 Fig — Amplification plot. (TIF) [file pone.0170399.s007.tif]

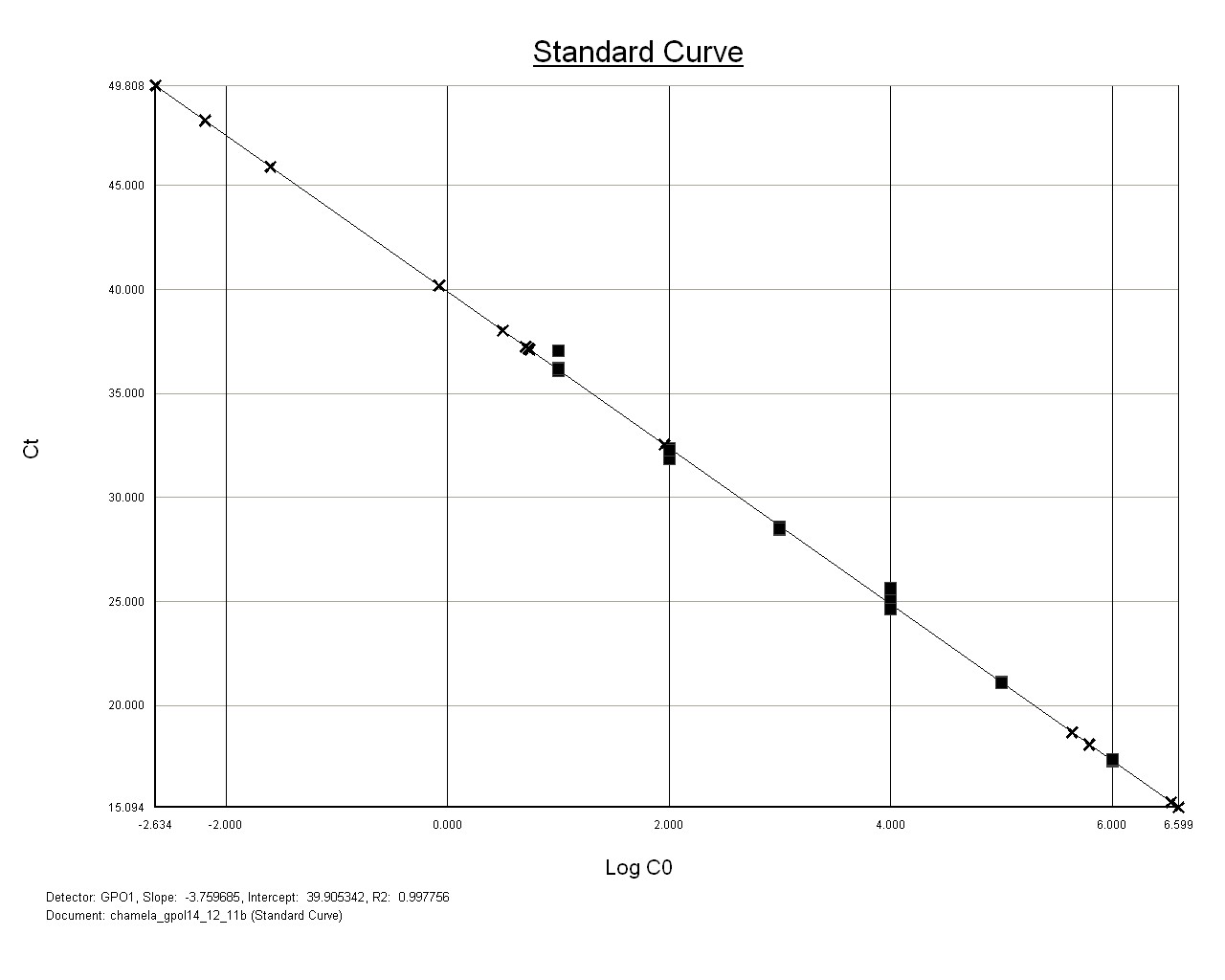

Supplement: S6 Fig — Standard curve. (TIF) [file pone.0170399.s008.tif]

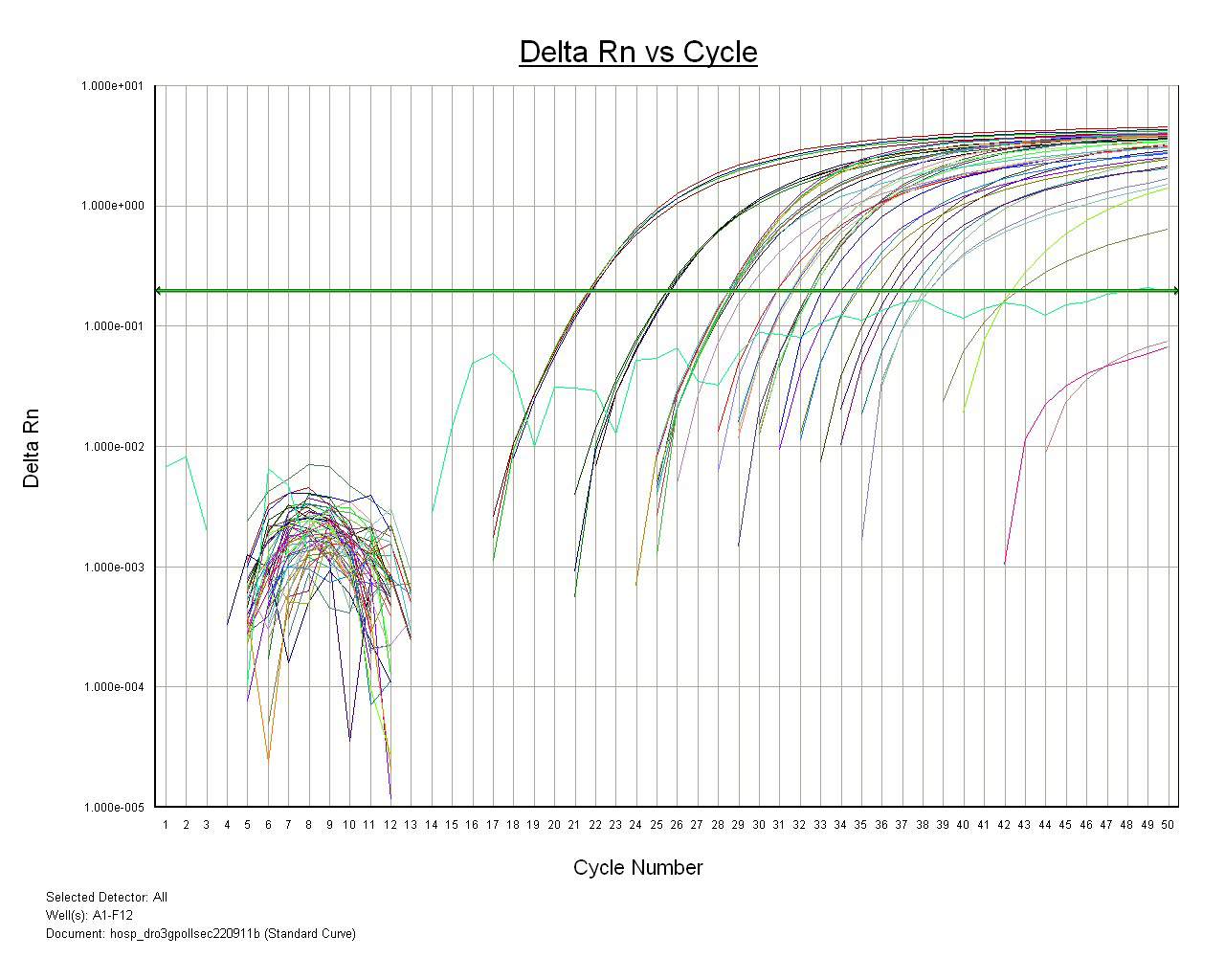

Supplement: S7 Fig — Amplification plot. (TIF) [file pone.0170399.s009.tif]

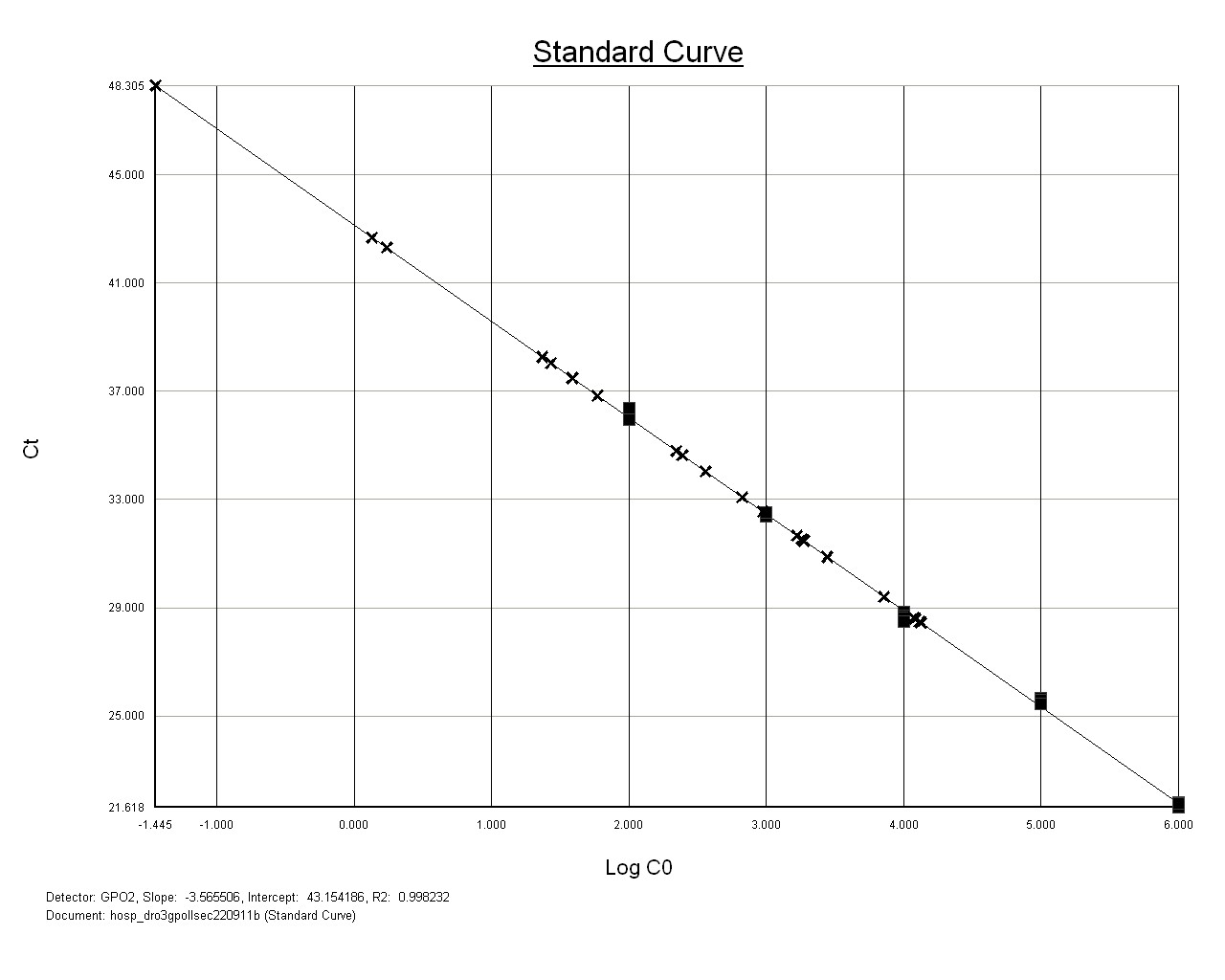

Supplement: S8 Fig — Standard curve. (TIF) [file pone.0170399.s010.tif]

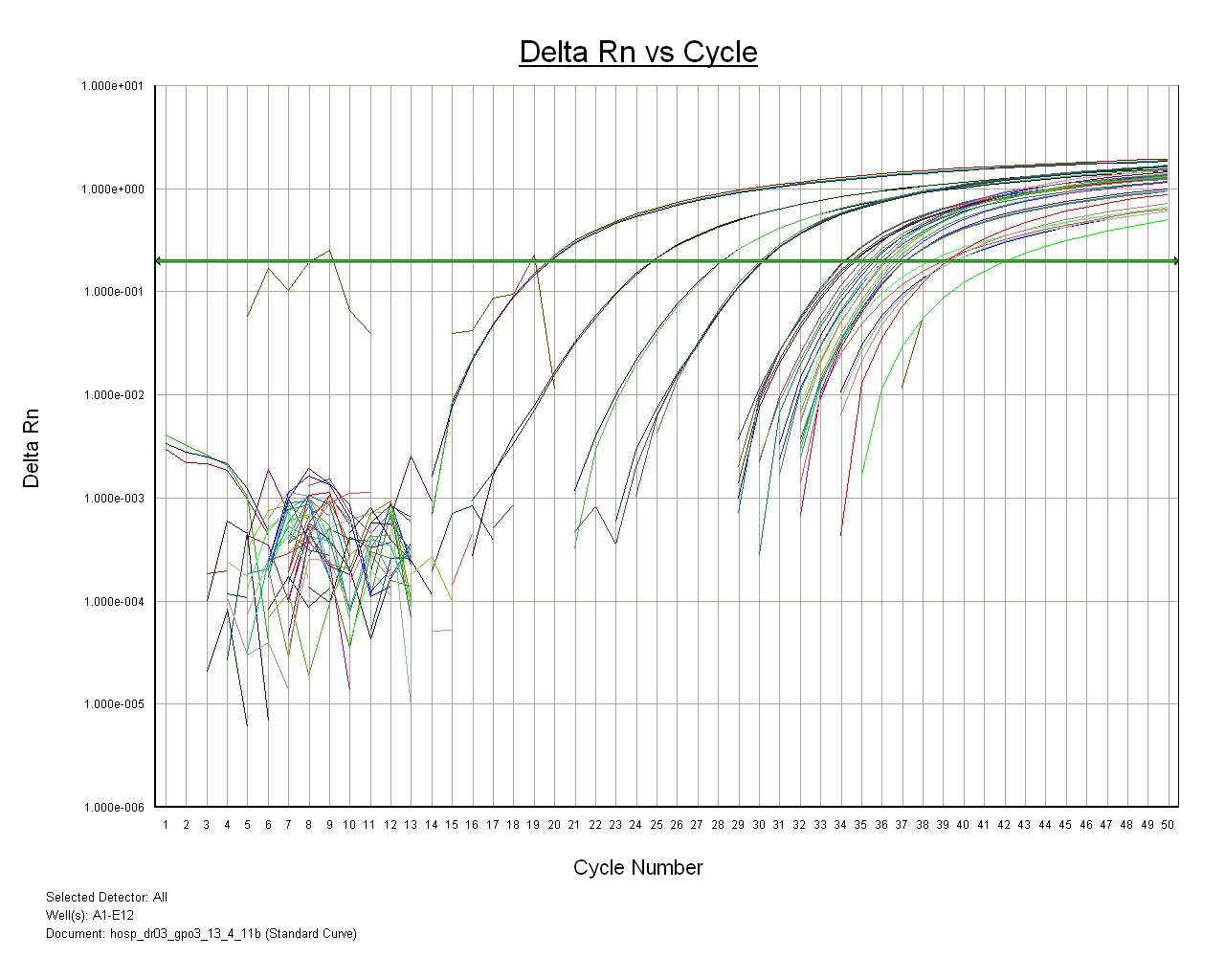

Supplement: S9 Fig — Amplification plot. (TIF) [file pone.0170399.s011.tif]

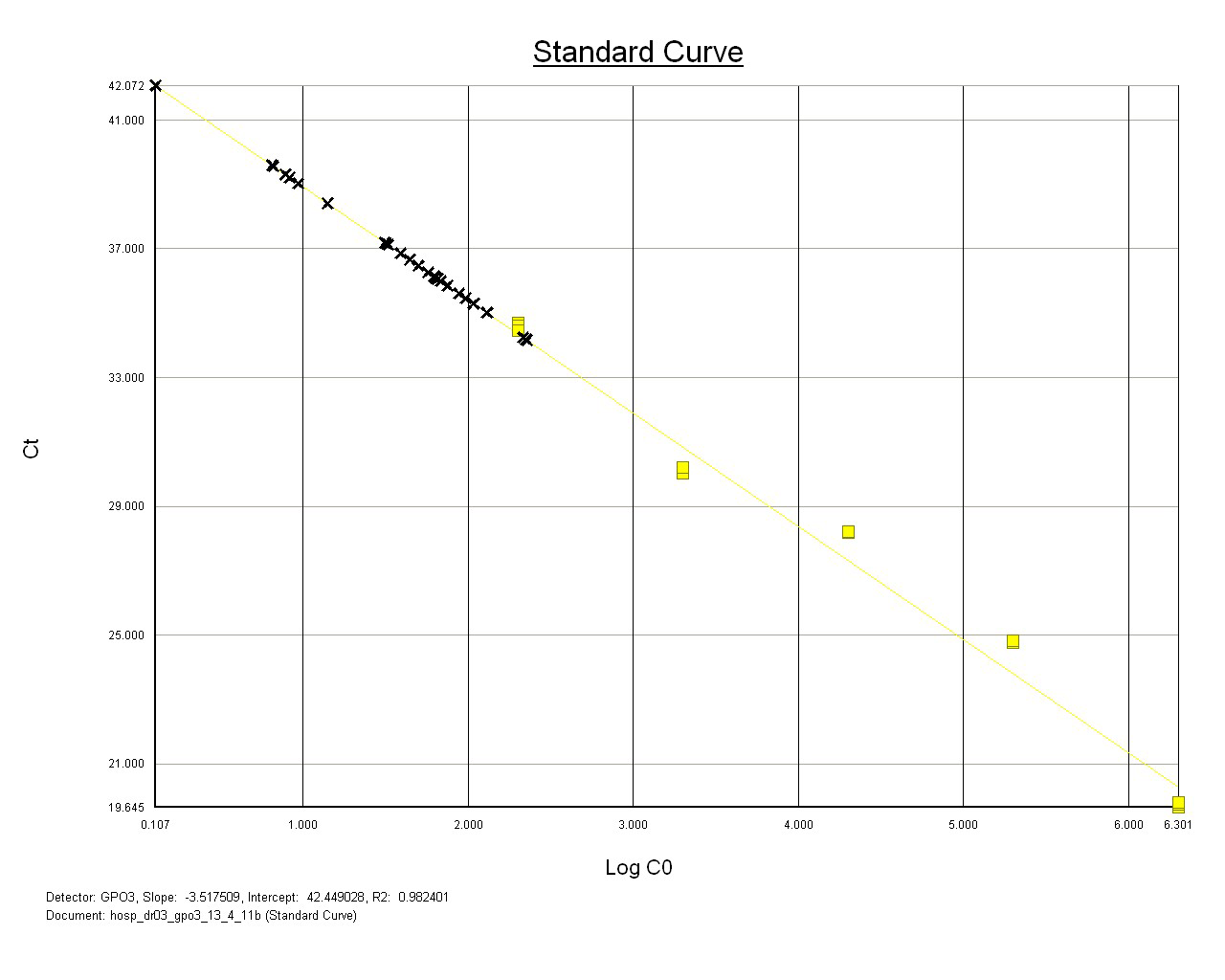

Supplement: S10 Fig — Standard curve. (TIF) [file pone.0170399.s012.TIF]

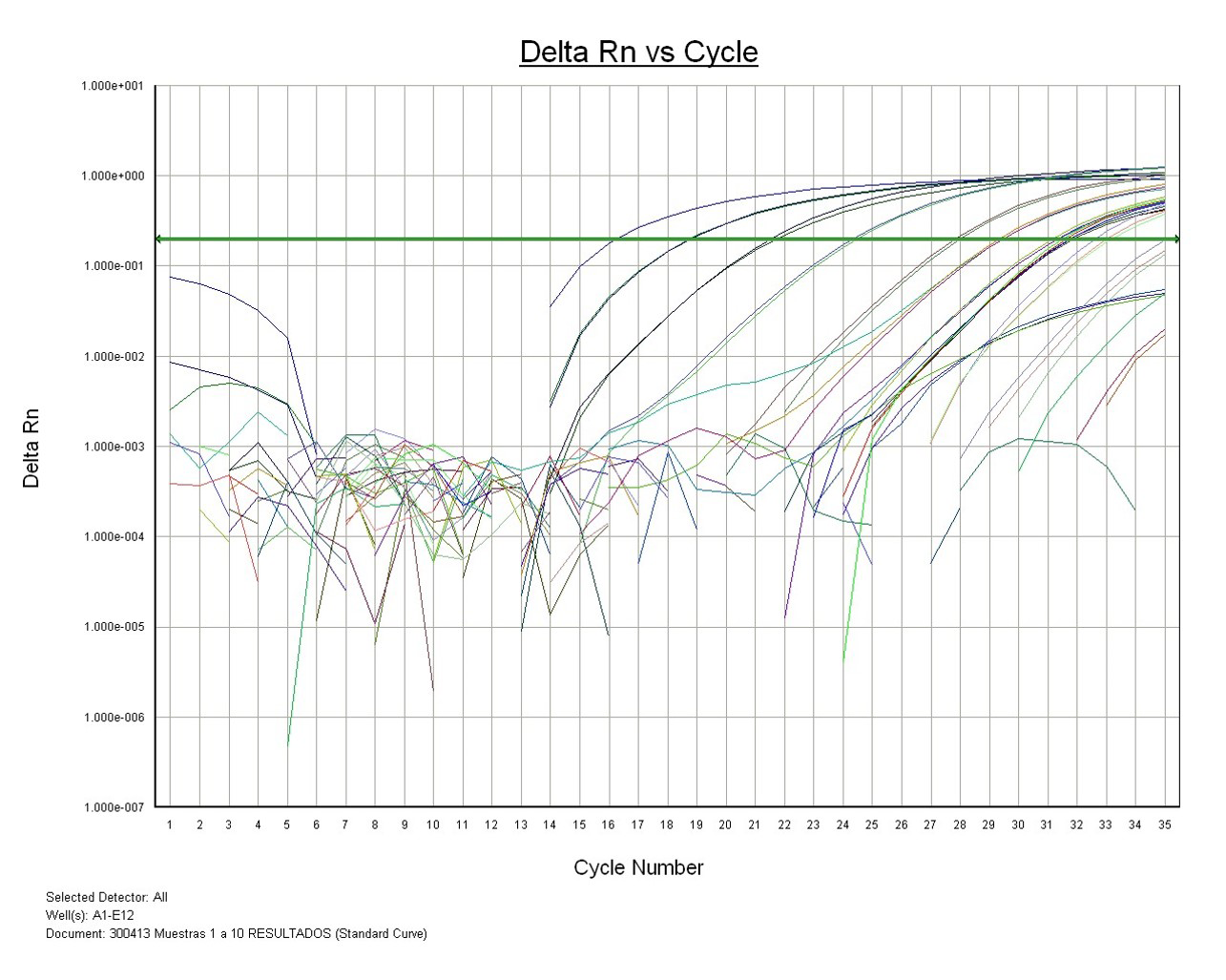

Supplement: S11 Fig — Amplification plot. (TIF) [file pone.0170399.s013.tif]

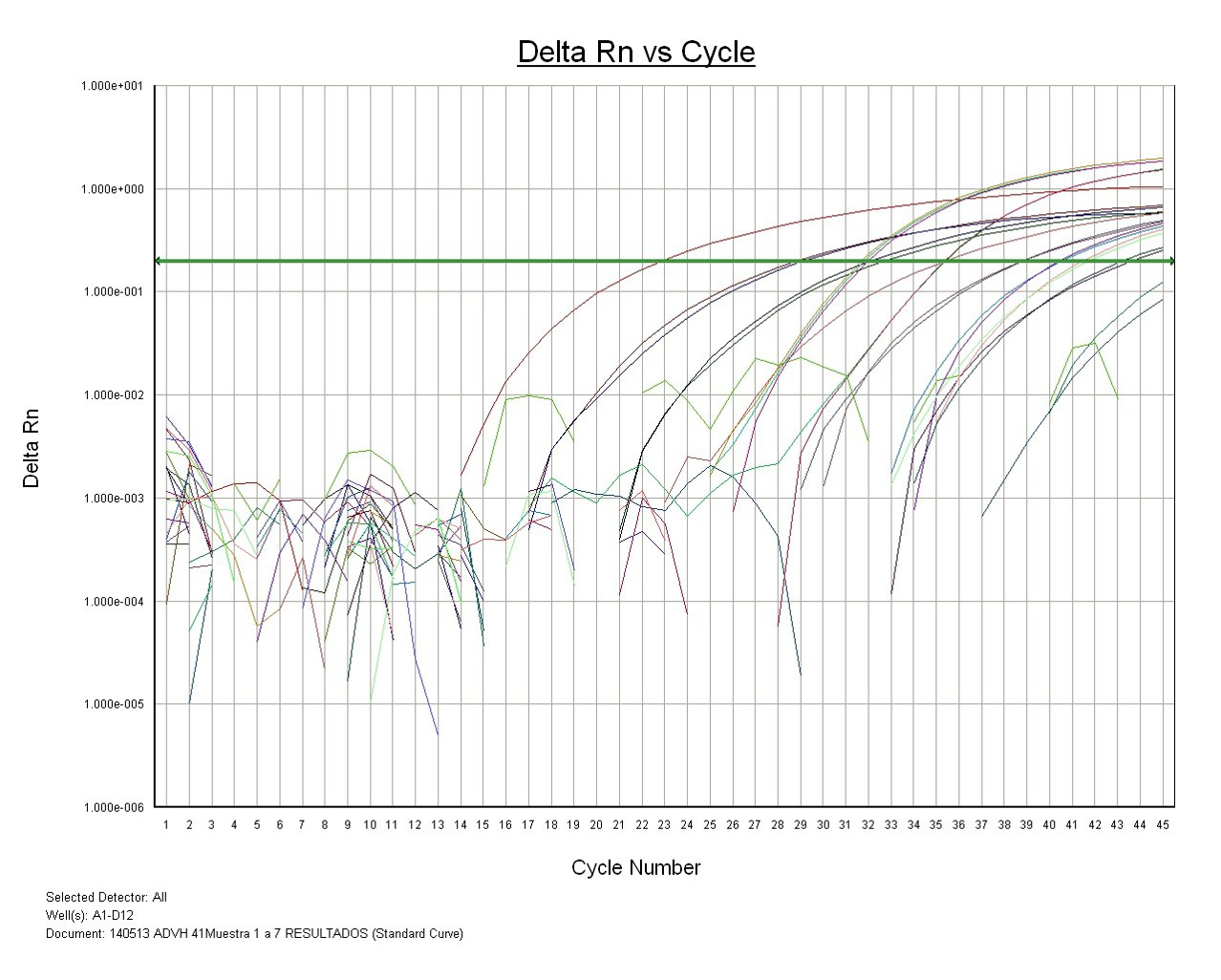

Supplement: S12 Fig — Amplification plot. (TIF) [file pone.0170399.s014.tif]
